# Abundance of *Ixodes ricinus* and prevalence of *Borrelia burgdorferi* s.l. in the nature reserve Siebengebirge, Germany, in comparison to three former studies from 1978 onwards

**DOI:** 10.1186/1756-3305-5-268

**Published:** 2012-11-21

**Authors:** Alexandra Schwarz, Václav Hönig, Zuzana Vavrušková, Libor Grubhoffer, Carsten Balczun, Antje Albring, Günter A Schaub

**Affiliations:** 1 Institute of Parasitology, Biology Centre, Academy of Sciences of Czech Republic, České Budĕjovice, Czech Republic; 2 Group Zoology/Parasitology, Ruhr-Universität Bochum, Bochum, Germany; 3 Faculty of Science, University of South Bohemia, České Budĕjovice, Czech Republic

**Keywords:** *Ixodes ricinus*, Tick density, *Borrelia* prevalence, *Borrelia lusitaniae*, Multiple infections, Siebengebirge

## Abstract

**Background:**

During the last decades, population densities of *Ixodes ricinus* and prevalences of *Borrelia burgdorferi* s.l. have increased in different regions in Europe. In the present study, we determined tick abundance and the prevalence of different *Borrelia* genospecies in ticks from three sites in the Siebengebirge, Germany, which were already examined in the years 1987, 1989, 2001 and 2003. Data from all investigations were compared.

**Methods:**

In 2007 and 2008, host-seeking *I. ricinus* were collected by monthly blanket dragging at three distinct vegetation sites in the Siebengebirge, a nature reserve and a well visited local recreation area near Bonn, Germany. In both years, 702 ticks were tested for *B. burgdorferi* s.l. DNA by nested PCR, and 249 tick samples positive for *Borrelia* were further genotyped by reverse line blotting.

**Results:**

A total of 1046 and 1591 *I. ricinus* were collected in 2007 and 2008, respectively. In comparison to previous studies at these sites, the densities at all sites increased from 1987/89 and/or from 2003 until 2008. Tick densities and *Borrelia* prevalences in 2007 and 2008, respectively, were not correlated for all sites and both years. Overall, *Borrelia* prevalence of all ticks decreased significantly from 2007 (19.5%) to 2008 (16.5%), thus reaching the same level as in 2001 two times higher than in 1987/89 (7.6%). Since 2001, single infections with a *Borrelia* genospecies predominated in all collections, but the number of multiple infections increased, and in 2007, for the first time, triple *Borrelia* infections occurred. Prevalences of *Borrelia* genospecies differed considerably between the three sites, but *B. garinii* or *B. afzelii* were always the most dominant genospecies. *B. lusitaniae* was detected for the first time in the Siebengebirge, also in co-infections with *B. garinii* or *B. valaisiana.*

**Conclusions:**

Over the last two centuries tick densities have changed in the Siebengebirge at sites that remained unchanged by human activity since they belong to a nature reserve. Abiotic and biotic conditions most likely favored the host-seeking activity of *I. ricinus* and the increase of multiple *Borrelia* infections in ticks. These changes have led to a potential higher risk of humans and animals to be infected with Lyme borreliosis.

## Background

Ticks are obligate hematophagous ectoparasites and important vectors of infectious diseases transmitting parasites of livestock and humans, e.g. the etiologic agents of babesiosis, theileriosis or anaplasmosis and of human tick-borne encephalitis and Lyme disease [1,2]. The sheep tick *Ixodes ricinus* is the most common tick species and principal vector for various infectious diseases in Europe and some regions of Asia and North Africa [3]. The distribution and abundance of *I. ricinus* depends on various abiotic and biotic factors such as the microclimate, habitat (vegetation) and host cenosis [4]. The host-seeking activity of *I. ricinus* is favored by air temperatures between 7°C and 24°C and relative humidities of 45-100% due to the risk of desiccation [5,6]. Ecosystems that have a strong buffering capacity, for example, for humidity, such as mixed deciduous and coniferous forests with well-developed leaf and shrub layers are preferred tick habitats [7,8]. However, also forest biotopes differ in the abundances of *I. ricinus*, presumably correlated to the water content of the soil [7,8].

Since the development and survival of ticks strongly depends on climatic conditions, the distribution and abundance of ticks might also be influenced by global warming. In Sweden, the distribution of *I. ricinus* extended towards the north, and this was suggested to be caused by increased air temperatures which favored the survival, activity and development of ticks [4]. Similarly, in the Czech Republic *I. ricinus* spread towards higher altitudes, from 700m to 1100m above the sea level within the last 20 years [9].

In addition to climatic factors, the host cenosis also affects the distribution and abundance pattern of ixodid ticks. *I. ricinus* is an euryphage species that has a broad host spectrum and thus feeds on more than 300 vertebrate species [10]. It predominantly infests small rodents (mice), passerine birds and larger mammals such as hedgehogs, hares, squirrels, wild boar and roe deer [11]. Increased population densities of these hosts induce an increase in the densities of ticks (summarized by [8]). In addition, also the anthropogenic impact on habitat changes the temporal and spatial pattern of tick populations [12].

An increase in the abundance of *Ixodes* can increase the transmission risk of diseases, e.g. of the spirochete bacterium *Borrelia burgdorferi* sensu lato, the etiologic agents of Lyme disease which is endemic in Europe [13]. Prevalences of *B. burgdorferi* s.l. in *I. ricinus* range up to 11%, 43% and 58% in larvae, nymphs and adults, respectively [14]. During the last decades, *Borrelia* prevalences have increased in different regions in Europe, such as in Denmark and Germany [15,16]. Infection prevalences differ between different regions in Europe because the transmission of *Borrelia* depends on a complex zoonotic cycle between their reservoir hosts and their tick vectors. More than 50 avian and mammalian hosts are reservoir hosts for *B. burgdorferi* s.l. in Europe [17].

Different genospecies have been identified in the *B. burgdorferi s.l.* complex. In Europe, seven genospecies are prevalent, *B. burgdorferi* sensu stricto, *B. afzelii*, *B. garinii*, *B. valaisiana*, *B. bissettii, B. spielmanii*, *B. lusitaniae*[18,19], and *B. bavariensis,* a recently classified genospecies [20] that was previously described as the rodent-associated *B garinii* OspA serotype 4 [21]. Usually *B. burgdorferi* s.s., *B. afzelii* and *B. garinii* (including *B. bavariensis*) are present in tissues of Lyme disease patients [22,23]. Spirochetes of *B. valaisiana* were isolated from a few patients who showed erythema migrans or acrodermatitis chronica atrophicans manifestations and an old man who showed strong clinical evidence of neuroborreliosis [23,24]. *B. bissettii* was detected in tissues from a few patients suffering from Lyme borreliosis in Slovenia and in the Czech Republic [25,26]. *B. spielmanii* was present in the skin of a few patients with erythema migrans [27]. Only once, *B. lusitaniae* was identified in a patient, but he showed symptoms that are untypical for the clinical manifestations of Lyme disease [28]. In *I. ricinus* from Slovakia, Latvia, Germany, Portugal and the United Kingdom, the most prevalent *Borrelia* genospecies were *B. afzelii*, *B. garinii* (including *B. bavariensis*) and *B. valaisiana* with overall prevalences of 39.3%, 21.2% and 12.8%, respectively [29].

Prevalence and the distribution of *Borrelia* genospecies strongly depend on the local host cenosis due to the host′s reservoir competence [30]. *B. afzelii* is mainly associated with rodents and *B. garinii* and *B. valaisiana* with birds [31]*.* Both, rodents and birds are competent reservoir hosts of *Borrelia burgdorferi* s.s. [32]. Rodents do also serve as reservoir hosts of *B. bissettii* and *B. bavariensis*[21,33] and the garden dormouse seems to be the main reservoir host of *B. spielmanii* in Central Europe [34]. *B. lusitaniae* is associated with birds and lizards [35,36]*.*

In the present investigation, we determined the abundance of *I. ricinus* and the prevalence of *Borrelia* and of the different genospecies in ticks in the Siebengebirge, a nature reserve and well visited local recreation area near Bonn, Germany. In 2007 and 2008, ticks were collected at three sites that represented different plant communities and possessed different population densities of *I. ricinus*. These sites have been already examined in previous investigations in the years 1987, 1989, 2001 and 2003 [8,15,37]. Thus, abundance and prevalence were compared with previous investigations.

## Methods

### Study area and tick collections of all studies since 1978

In 1987 and 1989, 2001, 2003, 2007 and 2008 host-seeking ticks (nymphs and adults) were collected in the nature reserve Siebengebirge, a forested and hilly region located south-east of Bonn [8]. As presented in Table 1, ticks were collected throughout the seasonal tick activity period in each study year in the Siebengebirge. In all years, exactly at the same sites in three plant communities, the Fraxino-Aceretum pseudoplatani, the Luzulo-Fagetum milietosum (*Athyrium filix-femina* variant) and the Galio-Fagetum typicum (considered as Melico-Fagetum typicum by [15,37,38]) were chosen for the tick collections [8,15,37,38]. A detailed description of the examined plant communities including a map of the exact study sites was previously published in Schwarz *et al.*[8]. Briefly, study site 1 (N50°39'37.1'',E 7°14'55.4'', 298m) in the Galio-Fagetum typicum (considered as Melico-Fagetum typicum by [15,37] and study field 2 by Schwarz et al. [8]) possessed a highly developed herb layer and well-developed shrub layer (dominant plant species, e.g. *Melica uniflora*) and a dry to wet soil water capacity [8]. Study site 2 (N50°39'53.2'', E7°13'05.8'', 130m, site 3 by [8]) in the Fraxino-Aceretum pseudoplatani was a dry to fresh vegetation type with a poorly developed herb and shrub layer (dominant plant species, e.g. *Dentaria bulbifera*). The soil water capacity was moderate. The third study site (N50°39'51.5'', E7°13'19.2'', 123m, site 4 by [8]) located in the Luzulo-Fagetum milietosum was the richest plant community in species and plant densities (dominant plant species, e.g. *Athyrium filix-femina*) and it had a high soil water capacity. In our previous study in 2003, medium, low and high numbers of ticks were characteristic for the Galio-Fagetum typicum, the Fraxino-Aceretum pseudoplatani and the Luzulo-Fagetum milietosum, respectively [8].

**Table 1:** **Overview of the methodological differences in the *Ixodes ricinus *studies in the Siebengebirge in 1987, 1989, 2001, 2003, 2007 and 2008**

**Methods**	1987/89*	**2001**	**2003**	**2007**	**2008**
Months^1^	Apr-Oct	May, Aug-Oct	May-Nov	May-Nov	May-Nov
Frequency	monthly	weekly	weekly	monthly	monthly
Size	100m^2^	100m^2^	225m^2^	100m^2^	100m^2^
Air temp.^2^	≥16°C	≥16°C	8°C-25°C	15°C-25°C	15°C-23°C
Humidity^2^	≥80%	≥80%	57%-74%	45%-85%	52%-85%
DNA extraction	n/a^3^	Ammonia solution	n/a	Ammonia solution	Chelex 100 resin solution
*Borrelia* detection	IFA^**+**^	Simple, modified PCR [45]	n/a	Nested PCR [44]	Nested PCR [44]
		Nested PCR [44]			
		Modified PCR [46]			
		IFA [15,38]			
*Borrelia* genotyping	n/a	Reverse line blotting^4^[44]	n/a	Reverse line blotting^5^[44,47]	Reverse line blotting^5^[44,47]

*The years 1987 and 1989 are listed together because the same methods were used in both study years for the study of *I. ricinus* abundances and *Borrelia* prevalences.

^1^ Tick collections were carried out in the respective months (Apr = April, Aug = August, Oct = October, Nov = November).

^2^Air temperatures and relative humidities were measured 5cm above the ground at the study sites in all years.

^3^n/a = not applicable.

^4^*B. burgdorferi* s.l., *B. burgdorferi* s.s., *B. garinii*, *B. afzelii* and *B. valaisiana* were identified by reverse line blotting according to Rijpkema et al. [44].

^5^*B. burgdorferi* s.l., *B. burgdorferi* s.s., *B. garinii* and *B. afzelii* were identified by reverse line blotting according to Rijpkema et al. [44] and DNA probes for *B. garinii*, *B. valaisiana*, *B. lusitaniae*, *B. spielmanii* and B*. bissettii* were designed according to Gern et al. [47].

^**+**^ IFA = Immunofluorescence assay.

In all Siebengebirge′s studies, ticks were collected monthly by repeated blanket dragging [8,37,38], with the exception of the study in 2001 when weekly tick collections were carried out [15] (Table 1). In all studies, a cotton flannel was used for blanket dragging, and the sites were repeatedly re-flagged until no more ticks were collected. According to Hubálek *et al*. [6], tick collections in 2003, 2007 and 2008 were carried out when air temperatures were between 7°C and 24°C and a relative humidity between 45% and 100% without rain and no strong winds occurred. The effective measured temperature and relative humidity ranges at the collection sites were listed for the three years in Table 1. In the years 1978, 1989 and 2001 ticks were collected when at least 16°C and 80% relative humidity was reached without rain and no strong winds [15,37,38]. All collected ticks were preserved in 70% ethanol and identified to species level in the laboratory [39].

### DNA extraction

In a chronological arrangement of the methodologies, in 2008 all adults and up to about 60 nymphs/month/site (in total 61 adults and 641 nymphs) were homogenized each in 200 μl 20% Chelex 100 resin solution (Bio-Rad) using the TissueLyser II (Qiagen) and stainless steel beads (5 mm) [40,41]. For total DNA extraction, 120 μl of each homogenate were incubated at 56°C overnight, vortexed and incubated for 10 min at 96°C. After centrifugation at 15,000 g for 3 min, the supernatant was used directly for PCR.

In 2007, all adults and up to about 90 nymphs/month/site (in total 50 adults and 652 nymphs) were pestled each in 100 μl 1.25% ammonia [42]. The tick homogenates were boiled at 100°C for 20 min, cooled down briefly, centrifuged at 16,000 g for 5 min and the supernatants boiled again to evaporate the ammonia until 30 μl of DNA solution was left.

In 2001, Kampen *et al.*[15] randomly selected 366 nymphs and 179 adults in 2001 for *Borrelia* examination. Similar to our study in 2007, the DNA of these ticks was extracted by ammonia (Table 1).

The following *B. burgdorferi* s.l. genospecies served as positive controls in PCR and/or reverse line blottings: *B. burgdorferi* sensu stricto N40, B31 and CB53, *B. garinii* PSth, *B. afzelii* VS461, *B. valaisiana* VS116 and *B. lusitaniae* PotiB3. The strains N40 and PSth were provided by the Baden-Wuerttemberg State Health Office, Stuttgart, Germany, B31 by J. F. Anderson (Connecticut Experiment Station, New Haven, USA), CB53 by J. Kopecký (Institute of Parasitology, ASCR, České Budĕjovice, Czech Republic) and the strains VS461, VS116 and PotiB3 by Ian Livey (Baxter Innovations, Orth an der Donau, Austria). All bacteria were cultured in BSK-H medium (Sigma-Aldrich) at 34°C as described previously [43].

Total DNA of the *B. burgdorferi* s.s. strain N40 and *B. garinii* PSth was isolated using Chelex 100 (Bio-Rad) [40]. Briefly, 100 μl of each *Borrelia* culture was centrifuged and the pellet resuspended in 40 μl of a 20% Chelex 100 suspension. The suspensions were incubated at 56°C for 30 min and, after thoroughly mixing, boiled for 10 min. Chelex 100 was removed by a final centrifugation step and the supernatant stored at −20°C. DNA of the *B. burgdorferi* s.s. strains B31 and CB53, *B. afzelii* VS461, *B. valaisiana* VS116 and *B. lusitaniae* PotiB3 were prepared using the DNeasy Blood and Tissue Kit (Qiagen) according to the manufacturer′s instructions.

### Detection of *B. burgdorferi* s.l

In 2008 and 2007, ticks were tested for *Borrelia* DNA by nested PCR according to Rijpkema *et al*. [44]. The first PCR mix contained 5 μl total DNA and final concentrations of 200 nM *B. burgdorferi* s.l. specific primers targeting the 5S-23S rDNA intergenic spacer region (23SN1 and 23SC1; [44]), 100 μM dNTPs, 1.5 mM MgCl_2_ and 1.25 U GoTaq Flexi DNA Polymerase (Promega). The following DNA amplification step using 35 cycles was set up: 94°C for 30 sec, 53°C for 30 sec and 72°C for 1 min. For the second PCR, 5 μl of the first PCR product using the same PCR mix and the specific primers 23SN1 and 5SCB without biotin label of the 5S-23S rDNA intergenic spacer region were used [44]. The same thermal cycling conditions of the first PCR were set up for the second PCR but using a primer annealing temperature of 55°C. Positive and negative controls were always included, and nested PCR products were screened for *B. burgdorferi* s.l. DNA by agarose gel electrophoresis.

In 2001 [15], *Borrelia* infection in ticks were analyzed by simple and nested PCR and immunofluorescence assay (IFA) according to Liebisch *et al*. [45] (with modifications), Rijpkema *et al.*[44] and Kurtenbach *et al.*[38]/Kampen *et al*. [15], respectively. A third, modified PCR protocol originally performed by Schwartz *et al*. [46] was additionally applied in 2001 if contradictory results between the simple and nested PCR approaches occurred.

In 1987, Kurtenbach *et al*. [38] examined 1189 nymphal and adult *I. ricinus* and 1050 nymphs and adults in 1989 for *Borrelia* infection. *Borrelia* prevalences were calculated without specification of the respective year; only prevalences of 1987/89 were published. The same IFA protocol as used by Kampen *et al*. [15] in 2001 was carried out for the tick examinations in 1987/89 [38].

### Genotyping of *Borrelia* species

A total of 249 tick samples positive for *B. burgdorferi* s.l. in 2007 and 2008 were further identified to the genospecies level [47]. Briefly, *B. burgdorferi* s.l. DNA was amplified by PCR using the 5′-biotinylated *Borrelia* specific B-5SBor primer and the 23SBor primer [48]. A touchdown PCR with an annealing temperature starting from 60°C to 52°C (1°C decrease per cycle) was set up to minimize amplification of non-specific DNA products. After the final annealing temperature was reached a further amplification step of 40 cycles using 52°C was carried out [49]. Amplification products were hybridized to 14 *Borrelia* specific oligonucleotide probes detecting the following genospecies [47]: *B. burgdorferi* s.l. (SL probe), *B. burgdorferi* s.s. (SS probe), *B. garinii* (GA, GANE and GANE1 probe), *B. afzelii* (AF probe), *B. valaisiana* (VSNE probe), *B. lusitaniae* (LusiNE, LusiNE1 and LusiNE2 probe), *B. spielmanii* (SpiNE2 and SpiNE3 probe) and *B. bissettii* (BisNE1 and BisNE2 probe). *B. burgdorferi* s.l., *B. burgdorferi* s.s., *B. garinii* and *B. afzelii* probes were designed according to Rijpkema *et al*. [44]. All other probes (including probes GANE and GANE1) were used according to Gern *et al*. [47]. Hybridized products were visualized by chemiluminescence using the ECL Detection Reagent and Hyperfilm ECL (GE Healthcare). Negative controls were included, and *Borrelia* DNA of the different genospecies served as positive controls. Additionally, *Borrelia* PCR products that hybridized with the probes GA, GANE1 and LusiNE1 were sequenced using OspA primers in order to distinguish between *B. garinii* and *B. bavariensis* genotypes as described previously [47,50].

Additionally, *Borrelia* samples from 2001 were genotyped by Kampen *et al.*[15] also using reverse line blotting. Similar to the studies in 2007 and 2008, probes for *Borrelia* identification targeted the 5S-23S rDNA spacer region. Probes were designed for the detection of *B. burgdorferi* s.l., *B. burgdorferi* s.s., *B. garinii*, *B. afzelii* and *B. valaisiana* according to Rijpkema *et al*. [44]*. B. lusitaniae, B. spielmanii* and *B. bissettii* identifications were not carried out in 2001.

### Data analysis

Tick densities and *Borrelia* prevalences were compared between the different studies in the Siebengebirge from 1987/89 to 2008. For tick density comparisons, only the months May to September were compared from each study year and the densities were calculated for 100m^2^ of study site. Therefore, the average monthly tick abundances from 2003 were recalculated from 225m^2^ to 100m^2^.

For the comparison of *Borrelia* prevalences throughout the different study years, IFA data from 1987/89 and 2001 were compared and nested PCR *Borrelia* data from 2001 with the results from 2007 and 2008 because in those years the same experimental protocols were used. In 2001, the same ticks were examined by IFA and nested PCR by Kampen *et al*. [15].

Statistical analysis of data was performed using Prism 4 (GraphPad Software). Differences in tick abundances, infection prevalences with *Borrelia* and *B. burgdorferi* s.l. genospecies between the three study sites and study years were analyzed by the chi square test or Mann–Whitney U test. Only *Borrelia* prevalences derived from more than 20 ticks were statistically compared. Climate parameters between different years of the last two centuries were compared by a one - way analysis of variance (One -Way ANOVA) with a pairwise multiple comparison procedure (Tukey test), the Kruskal-Wallis test or the Mann–Whitney U test. Correlations of tick densities with *Borrelia* prevalences were tested using the Spearman’s Rho rank correlation test. *P*-values of 0.05 or less were considered statistically significant for all tests.

## Results

### Abundances of *I. ricinus* in 2007 and 2008

**Figure 1:**
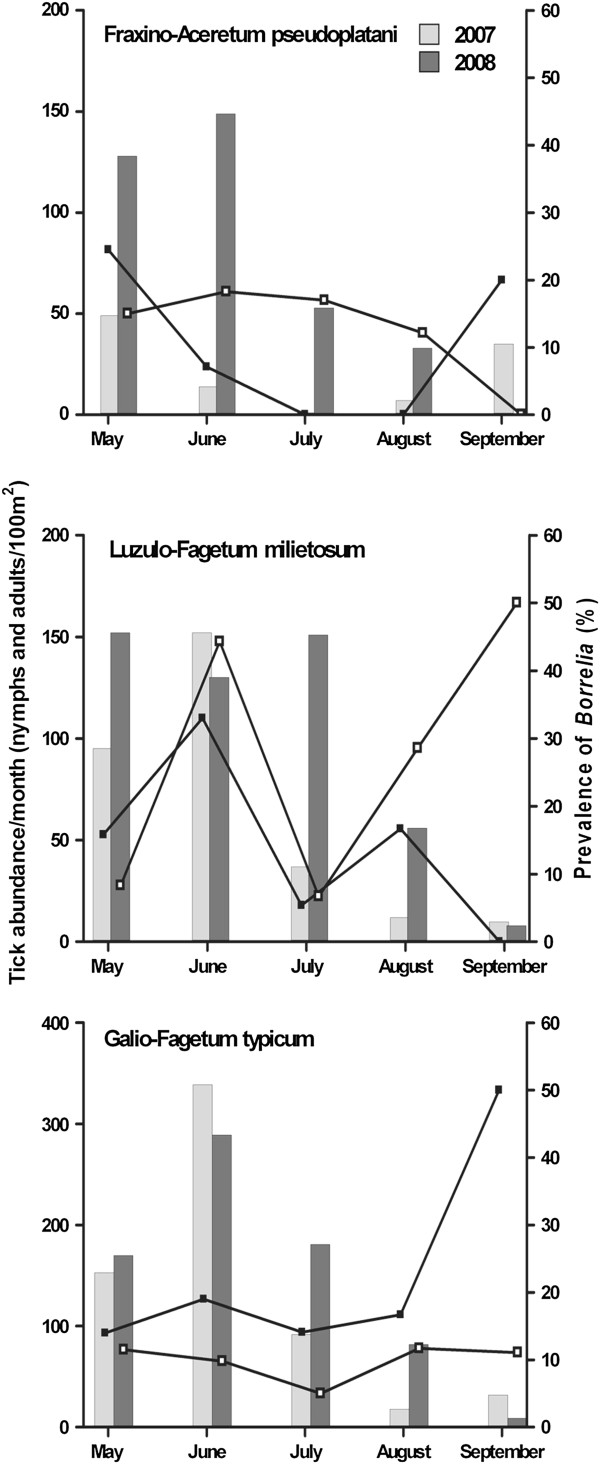
**Monthly abundances of *I. ricinus *and *Borrelia burgdorferi *s.l. prevalences at three study sites in the Siebengebirge in 2007 and 2008.** From May to September 2007 and 2008, host-seeking *I. ricinus* were collected by blanket dragging at three plant communities (Galio-Fagetum typicum, Fraxino-Aceretum pseudoplatani and Luzulo-Fagetum milietosum) in the nature reserve Siebengebirge, and the ticks were examined for *Borrelia* infection. The monthly abundances of ticks (columns) and *Borrelia burgdorferi* s.l. prevalences (line diagram) in 2007 (black squares) and 2008 (white squares) at the three study sites are presented in the figure.

Exclusively *I. ricinus* ticks were captured by blanket dragging. In 2007, a total of 1046 host-seeking ticks (50 adults, 996 nymphs) were collected (Table 2). In the Fraxino-Aceretum pseudoplatani, the plant community representing a low abundance biotope, the number of host seeking ticks decreased from May to July and increased up to September (Figure 1). In the other two plant communities, the Luzulo-Fagetum milietosum and the Galio-Fagetum typicum, that possessed higher numbers of ticks, the densities increased from May to June, decreased until August and increased slightly or remained at the same level in September (Figure 1). In 2008, a total of 1591 host-seeking *I. ricinus* (61 adults, 1530 nymphs) were collected at the three sites (Table 2). In the Fraxino-Aceretum pseudoplatani plant community more ticks were collected than one year before, and the monthly abundance graph showed a peak in June and no ticks in September (Figure 1). This was also evident for the site with the highest abundances, the Galio-Fagetum typicum, but at a higher abundance level, and a few ticks were collected in September (Figure 1). In the Luzulo-Fagetum milietosum, densities were similar in May, June and July and decreased until September (Figure 1).

**Table 2:** **Number of *Ixodes ricinus *and infection rates with *B. burgdorferi *s.l. at three plant communities in the Siebengebirge in 2007 and 2008**

		**2007**							**2008**						
**Plant community**	Tick stage*	**Total no. of ticks**	**No. of infected ticks/no. of examined ticks**					**Total infection rate (%)**	**Total no. of ticks**	**No. of infected ticks/No. of examined ticks**					**Total infection rate (%)**
			**Month**^**#**^							**Month**^**#**^					
			M	JN	JL	A	S			M	JN	JL	A	S	
Fraxino-Aceretum pseudoplatani	A	6	0/3	0/1	0/0	0/2	0/0	0	23	4/13	0/2	1/7	0/1	0/0	21.7
	N	100	12/46	1/13	0/1	0/5	7/35	20.0	340	5/47	11/58	8/46	4/32	0/0	15.3
Luzulo-Fagetum milietosum	A	18	1/4	0/12	0/2	0/0	0/0	5.6	15	0/8	0/0	0/6	1/1	0/0	6.7
	N	288	14/91	33/88	2/35	2/12	0/10	21.6	482	5/52	27/61	4/54	18/55	4/8	25.2
Galio-Fagetum typicum	A	26	0/9	1/6	1/6	0/2	2/3	15.4	23	1/8	2/9	0/1	1/5	0/0	17.4
	N	608	14/91	18/94	12/86	3/16	14/29	19.3	708	6/53	4/52	3/59	6/55	1/9	8.8
Total	A	50	5/50					10.0	61	10/61					16.4
	N	996	132/652					20.2	1530	106/641					16.5
	All	1046	137/702					19.5	1591	116/702					16.5

* The total number of collected adult (A) and nymphal (N) of *I. ricinus* at the three study sites are separately presented.

# Ticks were collected in May (M), June (JN), July (JL), August (A) and September (S) of 2007 and 2008 at the three study sites of the Siebengebirge.

Summarizing the numbers of ticks of all sites per year, population densities increased significantly from 2007 to 2008 (χ^2^ = 179.9, df = 4, p < 0.0001). Highest densities of 339 and 289 ticks/100m^2^ were found in the Galio-Fagetum typicum in June 2007 and 2008, respectively (Figure 1), but densities did not differ statistically significantly in direct comparison of the numbers in the Galio-Fagetum typicum and the Luzulo-Fagetum milietosum in 2007 (χ^2^ = 7.409, df = 4, p > 0.1). Excluding the lower numbers of collected ticks at all sites in September, densities increased from 2007 to 2008 up to 4.5-fold. This was caused by the enormous increase in the numbers of nymphal ticks since numbers of collected adults changed only slightly between the two years (Table 2).

### B. *burgdorferi* s.l. infection rates in 2007 and 2008

In 2007, 137 of 702 examined ticks (19.5%; 5 adults and 132 nymphs of 50 adults and 652 nymphs) were infected with *B. burgdorferi* s.l. (Table 2). In 2008, 116 of 702 ticks (10 adults and 106 nymphs of 61 adults and 641 nymphs) were infected, resulting in a prevalence of 16.5%. In 2007, at all collection sites, infection rates of nymphal ticks were significantly higher than that of adults (χ^2^ = 13.62, df = 2, p < 0.01, Table 2). However, in 2008 significantly more adults than nymphs were infected in the Galio-Fagetum typicum and Fraxino-Aceretum pseudoplatani sites (χ^2^ = 13.44, df = 2, p < 0.01), but the overall *Borrelia* prevalence in 2008 did not differ between nymphs and adults (Table 2).

In the Galio-Fagetum typicum, overall *Borrelia* prevalences were lower in 2007 than in 2008 (Mann–Whitney U test, p=0.0286) (Figure 1). At the other two sites, the infection rates did not differ statistically significantly between both years. In addition, *Borrelia* prevalences and tick densities were not correlated for all sites and both years (Spearman's rank correlation, r = −0.3163, p>0.05). In 2007 and 2008, 0–33 and 0–27 infected ticks/100m^2^ per month, respectively, were found in all plant communities. Excluding months representing prevalences based on <20 ticks, no strong differences in the prevalence between the different months were evident in the Fraxino-Aceretum pseudoplatani and Galio-Fagetum typicum for both years; only the latter showed an increase of the infection rate up to 50% in September 2007. In the Luzulo-Fagetum milietosum, in both years prevalences of *Borrelia* in June were about two and six times higher than in May and July.

### Distribution of *B. burgdorferi* s.l. genospecies in 2007 and 2008

**Figure 2:**
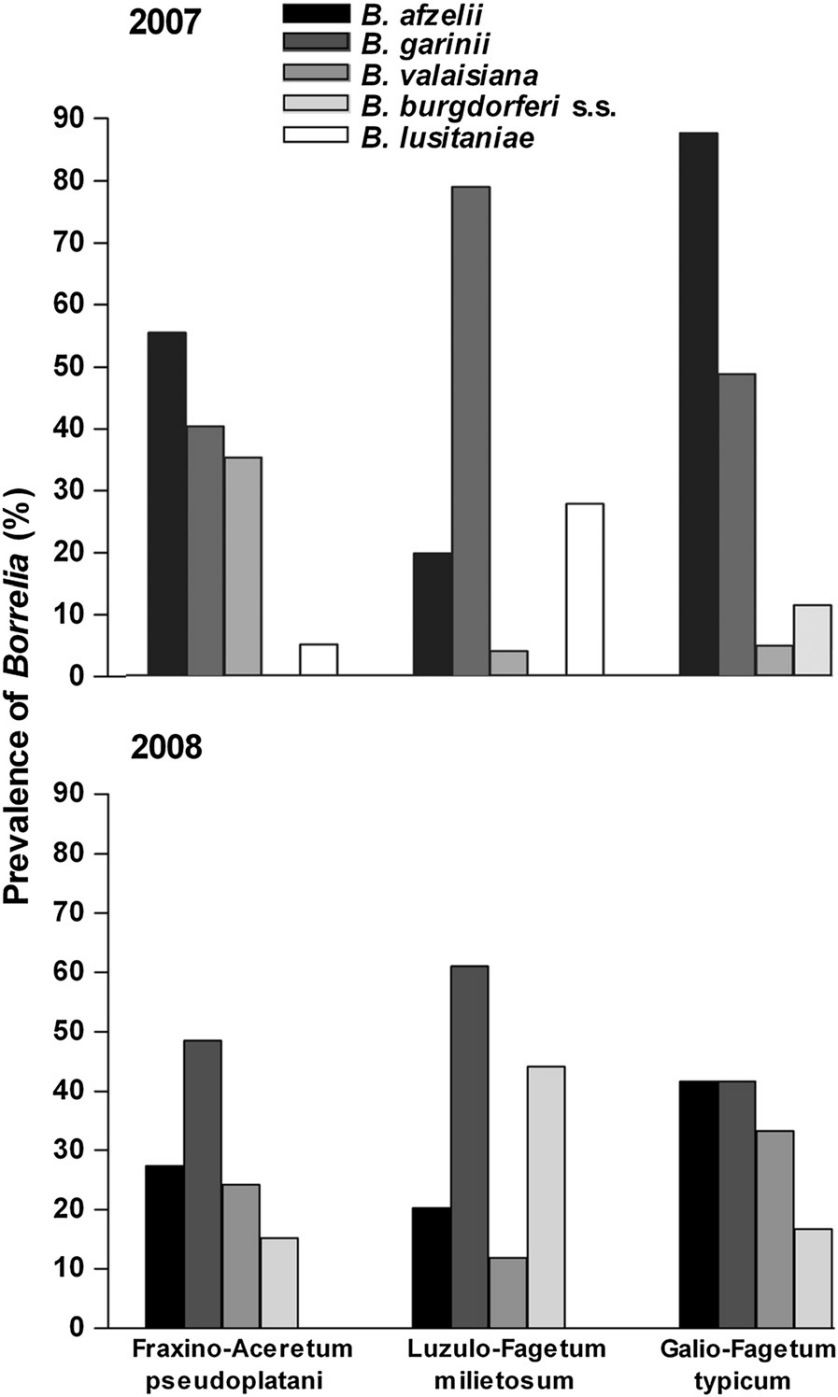
**Prevalence of different *Borrelia burgdorferi *s.l. genospecies in *I. ricinus *at the three plant communities in the Siebengebirge in 2007 and 2008.** Prevalences were calculated from the total number of infected ticks in the respective year and from the respective site used for the genospecies identification.

The ticks of the Siebengebirge were infected with five genospecies, *B. garinii, B. afzelii, B. valaisiana, B. burgdorferi* s.s. and *B. lusitaniae*. *B. spielmanii, B. bissettii* and *B. bavariensis* could not be detected at any site in both years. In 2007, the two most prominent genospecies in single and multiple *Borrelia* infections were *B. garinii* (58.6%) and *B. afzelii* (56.4%). Overall infection rates of the other genospecies were: 9.0% of *B. valaisiana,* 5.3% of *B. burgdorferi* s.s. and 11.3% of *B. lusitaniae*. The highest rates of *B. garinii*, 78.4%, occurred in the Luzulo-Fagetum milietosum and of *B. afzelii*, 87.1%, in the Galio-Fagetum typicum (Figure 2). *B. lusitaniae* appeared only at two sites and only in spring (apart from one infected tick in the Fraxino-Aceretum pseudoplatani in September) with a prevalence of 27.5% in ticks from the Luzulo-Fagetum milietosum, and *B. burgdorferi* s.s. only occurred in the Galio-Fagetum typicum (Figure 2).

In 2008, the genospecies composition differed significantly from that of 2007 (χ^2^ = 41.16, df = 4, p < 0.0001), but *Borrelia* infected *I. ricinus* also mainly contained *B. garinii* (overall infection prevalence at all sites including multiple infections: 53.4%). As in 2007, the highest prevalence with *B. garinii* occurred in the Luzulo-Fagetum milietosum (Figure 2). However, the overall infection prevalences of the other genospecies differed, with 26.7% for *B. afzelii,* 19.8% for *B. valaisiana* and 30.2% for *B. burgdorferi* s.s. At two sites *B. afzelii* prevalences were about 50% lower than in 2007*,* whereas *B. valaisiana* prevalences were higher in two plant communities (Figure 2). The strongest changes were evident for *B. burgdorferi* s.s. and *B. lusitaniae*. *B. burgdorferi* s.s. rates increased remarkably at two sites and this genospecies was detected for the first time in 2008 at all three sites. *B. lusitaniae* was not identified in 2008 in any plant community (Figure 2).

A comparison of the distribution of the different genospecies in nymphs and adults cannot be performed, since infections of only 15 adult ticks were identified; nine ticks with single infections covered one of the five genospecies (Table 3). Summarizing data of nymphs and adults for a comparison of single and multiple infections revealed the predominance of single *Borrelia* infections with *B. afzelii* or *B. garinii* in both years (Table 3). In 2007, both genospecies also predominated in six combinations of double infections (Table 3). The frequency of a specific *Borrelia* genospecies detected either as a single or a co-infection with other species did not differ significantly (χ^2^ = 0.05, df = 3, p > 0.05), e.g. 35 ticks were singly infected with *B. afzelii* and 40 ticks with this genospecies as well as at least one other *Borrelia* species in 2007. A high rate of multiple infections of 32.3% is only evident for the *B. afzelii/B. garinii* double infection in the Galio-Fagetum typicum. In this biotope, also the only type of a triple infection with *B. afzelii, B. garinii* and *B. burgdorferi* s.s. occurred. Additionally, *B. burgdorferi* s.s. was only detected in double and these triple infections of *I. ricinus*, not as a single infection in 2007 (Table 3).

**Table 3:** **Total number (No.) and percentage (%) of single and multiple infections of ticks with *Borrelia afzelii *(AF), *B. garinii *(GA), *B. valaisiana *(VA), *B. burgdorferi *s.s. (SS) and *B. lusitaniae *(LU) at the three collection sites**

**Plant community**	**Fraxino-Aceretum pseudoplatani**				**Luzulo-Fagetum milieotosum**				**Galio-Fagetum typicum**			
**Geno-**	**2007**		**2008**		**2007**		**2008**		**2007**		**2008**	
**species**	**No.**^**a**^	**%**^**b**^	**No.**^**a**^	**%**^**b**^	**No.**^**a**^	**%**^**b**^	**No.**^**a**^	**%**^**b**^	**No.**^**a**^	**%**^**b**^	**No.**^**a**^	**%**^**b**^
AF	6	30.0	5^1^	15.2	3	5.9	2	3.4	26	41.9	4	16.7
GA	4	20.0	13^3^	39.4	25	49.0	23	39.0	6	9.7	6^1^	25.0
VA	3	15.0	6	18.2	1	2.0	4	6.8	1	1.6	5^2^	20.8
SS	0	0.0	4	12.1	0	0.0	12^1^	20.3	0	0.0	2	8.3
LU	0	0.0	0	0.0	7^1^	13.7	0	0.0	0	0.0	0	0.0
AF/GA	3	15.0	2^1^	6.1	7	13.7	1	1.7	20^3^	32.3	2	8.3
AF/VA	2	10.0	1	3.0	0	0.0	1	1.7	1	1.6	2	8.3
AF/SS	0	0.0	1	3.0	0	0.0	4	6.8	4^1^	6.5	1	4.2
GA/VA	1	5.0	1	3.0	1	2.0	2	3.4	1	1.6	0	0.0
GA/SS	0	0.0	0	0.0	0	0.0	6	10.2	0	0.0	1^1^	4.2
GA/LU	0	0.0	0	0.0	7	13.7	0	0.0	0	0.0	0	0.0
VA/LU	1	5.0	0	0.0	0	0.0	0	0.0	0	0.0	0	0.0
AF/GA/VA	0	0.0	0	0.0	0	0.0	0	0.0	0	0.0	1	4.2
AF/GA/SS	0	0.0	0	0.0	0	0.0	4	6.8	3	4.8	0	0.0
Total	20	100	33	100	51	100	59	100	62	100	24	100

^a^ superscripts indicate the corresponding numbers of adult ticks with the respective infection.

^b^ calculated from the total number of infected ticks used for genospecies identification in the respective year and from the respective site.

In 2008, significantly more single than multiple infections with *B. garinii* predominated at three study sites (χ^2^ = 9.8, df = 3, p < 0.05) (Table 3). For *B. garinii* single infections ranged between 25.0% and 39.4% and for the other genospecies between 3.4% and 20.8% in the different plant communities. Double infections were present in only up to 10.2% of infected ticks, mainly including *B. garinii, B. afzelii* and *B. valaisiana.* At all sites, *B. burgdorferi* s.s. was detected as a single infection. Comparing double infections of both years *B. afzelii* and *B. garinii* appeared always in combination with three of the other four genospecies. Four ticks with triple infections in the Luzulo-Fagetum milietosum contained the same genospecies combination as the ticks in 2007 from the Galio-Fagetum typicum. In the latter biotope, a new combination with *B. afzelii, B. garinii* and *B. valaisiana* occurred in one tick.

### Climate conditions in the region of Bonn between 1987 and 2008

Climate records of the region of Bonn, near the Siebengebirge, revealed no striking differences in the mean monthly air temperatures between any year of 1987 until 2008 (One Way ANOVA, p > 0.05, Table 4). Furthermore, no statistically significant differences in the average monthly winter air temperatures from November to February before the collection periods in 1987, 1989, 2003, 2007 and 2008 were evident (Kruskal-Wallis test, p > 0.05, Table 4).

**Table 4:** Mean monthly air temperatures [°C] 2m above the soil near the Siebengebirge^a^ in different years

	**Jan**	**Feb**	**Mar**	**Apr**	**May**	**Jun**	**Jul**	**Aug**	**Sep**	**Oct**	**Nov**	**Dec**	**Year**	**Winter months**^**b**^
1986	2.6	−4.1	4.9	6.9	14.9	17.7	18.3	17.1	11.5	11.6	7.6	4.3	9.4	-
1987	−3.5	1.8	2.3	12.6	10.3	14.8	18.0	16.8	15.8	10.8	6.1	3.9	9.1	2.6
1988	5.8	4.0	4.9	9.4	15.9	20.6	17.4	17.7	14.1	11.1	5.2	6.5	11.1	-
1989	4.1	4.4	8.8	11.4	15.6	16.0	19.4	18.3	15.5	12.1	5.0	4.7	11.3	5.1
2002	3.2	7.0	7.3	9.7	14.3	18.0	17.9	18.8	13.9	10.0	8.3	3.9	11.0	-
2003	1.7	1.4	8.1	10.3	14.6	19.7	19.8	20.8	14.9	7.6	8.6	3.7	10.9	3.8
2006	0.2	1.8	3.8	9.1	14.5	17.8	23.3	16.2	18.4	14.0	8.9	5.8	11.2	-
2007	6.5	6.2	7.9	13.8	15.3	18.0	17.8	17.0	13.5	9.8	5.7	3.3	11.2	6.9
2008	5.9	5.1	5.8	8.8	16.5	17.3	18.2	18.2	13.0	10.2	6.5	2.0	10.6	5.0

^a^ Data were obtained from the weather station Cologne/Bonn (no. 10513) of the Deutscher Wetterdienst.

^b^ The mean temperature of the winter months were determined by calculating the mean monthly temperature from November of the previous year to February of the following year stated in the beginning of each line. For example, for 2007 the mean average temperature of the winter months was calculated from November 2006 until February 2007.

Precipitation levels in the region of Bonn during the tick collection months differed significantly between the collection years 1978, 1989, 2003, 2007 and 2008 (Kruskal-Wallis test, p<0.05) with high precipitation levels from May to August in 1987 and 2007 (Table 5). Significantly higher precipitation levels were also recorded in 2007 compared to 2003 (Mann–Whitney U test, p<0.05).

**Table 5:** Monthly precipitation heights [mm] near the Siebengebirge^a^ in different years

	**Jan**	**Feb**	**Mar**	**Apr**	**May**	**Jun**	**Jul**	**Aug**	**Sep**	**Oct**	**Nov**	**Dec**	**Year**	**Summer months**^**b**^
1987	59.6	68.9	104.0	39.0	70.6	123.0	126.8	83.3	50.2	38.2	80.6	42.3	73.9	100.9
1988	72.3	68.1	160.0	31.0	19.2	49.9	134.8	27.1	52.2	58.2	85.9	112.1	72.6	57.8
1989	27.6	57.1	65.8	103.1	26.3	61.1	49.9	67.9	37.8	62.6	38.4	95.1	57.7	51.3
2002	56.3	122.0	58.9	75.1	44.8	43.9	89.4	97.2	29.4	89.9	93.5	90.5	74.2	68.8
2003	77.1	29.2	43.5	43.4	55.2	72.7	53.4	55.3	50.8	95.1	45.6	54.0	56.3	59.2
2006	25.3	64.6	79.3	68.4	99.3	37.5	17.0	96.5	32.8	38.0	85.0	55.9	58.3	62.6
2007	82.4	59.8	53.5	1.6	129.6	105.8	127.8	193.0	59.9	33.2	70.8	52.2	80.8	139.1
2008	35.6	38.5	71.0	64.0	36.6	89.8	131.7	62.9	52.1	80.4	45.1	55.9	63.6	80.3

^a^ Data were obtained from the weather station Cologne/Bonn (no. 10513) of the Deutscher Wetterdienst.

^b^ The mean precipitation heights of the summer months were obtained by calculating the monthly precipitation from May to August of the same year.

## Discussion

### Comparison of tick densities and climate conditions between the different study years

**Figure 3:**
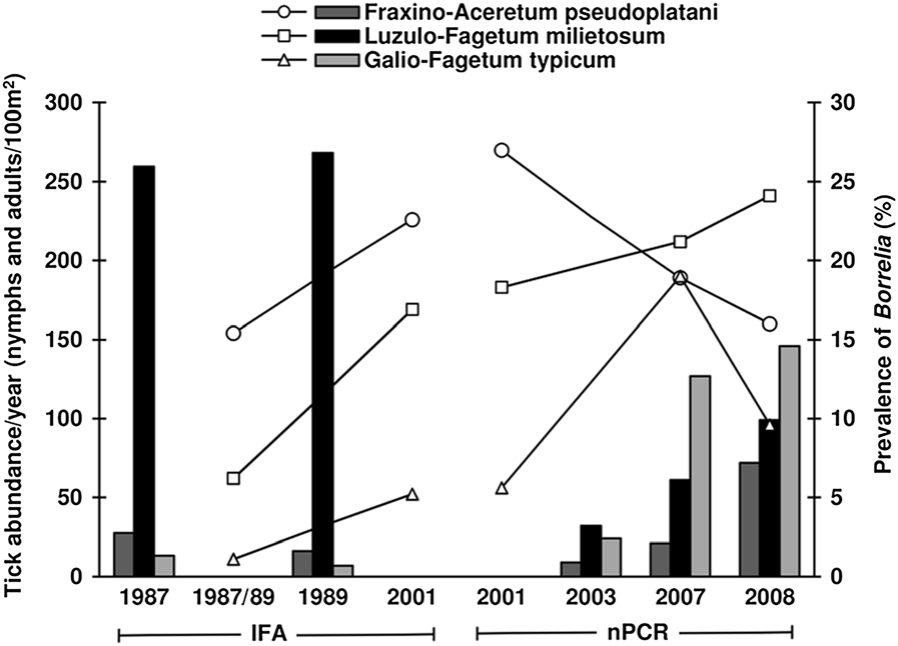
**Abundances of ticks at three plant communities in the Siebengebirge and *Borrelia burgdorferi *s.l. prevalences from 1987 to 2008.** Abundances are presented as columns: Fraxino-Aceretum pseudoplatani (dark grey), the Luzulo-Fagetum milietosum (black) and the Galio-Fagetum typicum (light grey). Monthly tick abundances per 100m^2^ of study site from May to September of each study year were calculated; in 2001 tick densities were not determined [15]. *Borrelia* prevalences for the Fraxino-Aceretum pseudoplatani, the Luzulo-Fagetum milietosum and the Galio-Fagetum typicum, are presented as circles, squares and triangles, respectively. Tick samples from 1987/89 and 2001 were examined for *Borrelia* infection by the immunofluorescence assay (IFA) using the same experimental protocol [15,38]. The same ticks used for IFA in 2001 were also analyzed for *Borrelia* infection by nested PCR (nPCR) [15]. Additionally, the same nPCR approach from 2001 was employed to analyze *I. ricinus* in 2007 and 2008. Accordingly, *Borrelia* prevalences from 1987/89 were compared with results from 2001, and 2001 prevalence data were compared with the data from 2007 and 2008. *Borrelia* infection rates were not determined in 2003 [8].

Long-term investigations on the distribution of the tick *I. ricinus* and on *Borrelia* infection rates in these ticks are rare, and in Germany these investigations have been only performed since 1987 in the nature reserve Siebengebirge, a very popular recreation area of the Bonn-Cologne region. The nature reserve possesses a very species-rich vegetation with approximately 100 different plant communities [51] which support the development of *I. ricinus* differently [8]. According to a study monitoring tick densities including Geographic Information Systems (GIS) in 2003, 57% of the total area of the nature reserve possesses very high (≥51 ticks/100m^2^) to medium tick densities (11–40 ticks/100m^2^) [8]. Comparing exactly the same sites examined since 1987, tick densities changed considerably between 1987/89 and 2008 (Figure 3). In the Fraxino-Aceretum pseudoplatani (covering 0.3% of the nature reserve) the number of ticks/year/100m^2^ decreased from 1987 until 2003 to a minimum of 9 ticks/100m^2^ in 2003 and increased about 8-fold until 2008. In the Luzulo-Fagetum milietosum which covers 3% of the total area of the nature reserve (24% including all Luzulo-Fagetum sub-associations), the tick population density enormously decreased between 1987 and 2003, but then increased continuously to a 3-fold higher density compared to 2003. In the Galio-Fagetum typicum, the abundance increased continuously from 13 ticks/100m^2^ in 1987 to 146 ticks/100m^2^ in 2008. An assessment of the tick numbers for the entire Siebengebirge according to the GIS evaluation of *I. ricinus* in the Siebengebirge by Schwarz *et al.*[8] suggested that the increase in tick numbers may have a huge impact on the total number of ticks in the entire Siebengebirge because the Galio-Fagetum typicum is the third largest plant community, covering 10% of the total area of the nature reserve [52]. However, for example, differences in the host cenosis at the same plant communities in different areas of the Siebengebirge can change the distribution of tick populations.

Temperature is one of the most important abiotic factors for tick development [5,53,54], and global warming during the last two decades of the 20^th^ century is suggested to be one of the reasons for increasing tick abundances in Sweden or Great Britain [4,55]. Ticks were collected at similar air temperature conditions from May to September in all years [8,15,38] with a minimum recorded temperature of 14.7°C in August 2007 in the Luzulo-Fagetum milietosum (data not shown). Additionally, air temperatures of the region of Bonn did not significantly differ between the years. However, in comparison to other tick collection seasons relatively high air temperatures from November 2006 until April 2007 (8.9°C-13.9°C) were recorded that may have caused a higher survival rate of ticks in 2006 as well as a stronger increase of tick population densities in spring 2007 and thus an overall increase in abundances during the entire tick season in 2007.

In addition to temperature, humidity affects the development of ticks. The relative humidity at the three sites and during the tick collections differed between the years [8,15,38]. In 1987, 1989 and 2001, ticks were collected at a minimum of 80% relative humidity at the sites, and in the years 2003, 2007 and 2008 minimum relative humidities of 57% [8], 45% and 52% (Table 1), respectively, were recorded at the biotopes. During all 15 tick collections in 2003 less than 70% relative humidity occurred at all field sites [52], and in 2007 only in 7 out of 15 collections more than 70% relative humidity was recorded (data not shown). In 2008, a minimum of 70% relative humidity was reached during 6 out of 15 tick collections. Although from 2003 to 2008 in 62% of all collections drier climatic conditions occurred compared to 1987 and 1989, higher tick abundances were determined in some of the biotopes between 2003 and 2008 (Figure 3). The saturation deficit that depends on the air temperature and the relative humidity influences the questing behavior of ticks [5,56]. Therefore, numbers of questing ticks increased until a certain limit of the saturation deficit [57]. This may explain the increase in questing ticks at the different study sites from 2003 to 2008. However, the high air temperatures in summer 2003 most likely corresponded with the lower yearly tick densities in the Fraxino-Aceretum pseudoplatani and Luzulo-Fagetum milietosum (Figure 3), because the saturation deficit was very high at these sites, and a high saturation deficit can rapidly decrease the numbers of questing ticks [56].

In a correlation of abiotic factors and tick abundances, the number of host seeking ticks rose significantly with rising soil water content in 2003 [8] and that may also explain the increase in tick numbers in 2007. Comparing all three different plant communities, the Fraxino-Aceretum pseudoplatani soil had the lowest water content and this correlated with low numbers of collected ticks [8]. The soil water content is affected by the precipitation. Overall higher precipitation levels in the region of Bonn in 2007 compared to 2003 combined with the higher air temperatures during the winter and spring may explain the higher tick numbers in 2007 in comparison to 2003. In addition, the soil water content of the different sites was 1–2 times higher in May and June 2007 (data not shown) than in the dry summer 2003. Furthermore, the optimal abiotic conditions in summer 2007 most likely caused a strong increase in the density of the tick population which was not strongly reduced by the mild winter 2007/2008 (Table 4) and resulted in higher numbers of questing ticks in 2008, respectively. These data indicate an effect of global warming on the number of ticks, but a continuous monitoring of tick densities covering several years and a determination of soil water contents are necessary for a better conclusion.

### Comparison of *Borrelia* prevalences between the study years

*Borrelia* infection rates were sometimes positively correlated with tick densities [58,59]. However in the Siebengebirge, the prevalences of *Borrelia* decreased in the Fraxino-Aceretum pseudoplatani and the Galio-Fagetum typicum from 2007 to 2008, whereas the total number of ticks increased in these biotopes (Figure 3). Only the Luzulo-Fagetum milietosum showed consistently increasing infection rates of ticks with *Borrelia* from 1987 to 2001 (IFA data comparison) and from 2001 to 2008 (nested PCR data) as well as increasing tick abundances since 1987 (apart from the exceptional high abundances in that biotope in 1987 and 1989) [8,15]. Since climate factors cannot explain this phenomenon, biotic factors should be considered. Tick populations strongly depend on their hosts and infections with *Borrelia* can only be obtained from hosts [60-62]. Rabbits, foxes, roe and red deer, wild boar, mice and voles are abundant in the Siebengebirge and hosts of *I. ricinus*. Of these, *Apodemus sylvaticus*, *A. flavicollis* and *Clethrionomys glareolus* have been confirmed as reservoir hosts of *Borrelia* in the nature reserve [37]. Reservoir hosts of *Borrelia* differ considerably in their competence to acquire the infection and to enable a multiplication of the spirochetes for a successful transmission [61]. The reservoir potential of *Apodemus* spp. and *C. glareolus* differed even between biotopes in the Siebengebirge [37] which may be caused by differing host immune responses, for example by tick density-dependent resistance of the host against tick feeding. Another reservoir host for *B. burgdorferi* s.l. is the wild boar [63-66]. In the Siebengebirge, the numbers increased enormously during the last 50 years [67], but hunted boars were not strongly infested by ticks [68,69]. Much stronger infestation was seen in roe deer, which are abundant in the Siebengebirge, thus supporting tick populations [70]. However, roe deer are not a competent host for *B. burgdorferi* s.l. [71,72], most likely resulting in a so called dilution effect of *Borrelia*[73].

Such investigations are also required to explain the increased number of multiple infections with *Borrelia* in *I. ricinus* from the Siebengebirge. In 2007, significantly more ticks possessed multiple infections than in 2001 (χ^2^ = 7.7, df = 2, p < 0.05) [15]. In 2001, only seven double infections with *Borrelia* were detected [15], whereas 7-fold and 4-fold more multiple infections were recorded in 2007 and 2008, respectively. Increased numbers of ticks within the last few years may have given rise to the probability of more ticks co-feeding on the same host and this may have led to an increased exchange of different genospecies between host and ticks resulting in a higher burden of different *Borrelia* genotypes per tick and host [74] However, the lower percentages of double infections in the Siebengebirge in 2008 in comparison to 2007 indicate the existence of specific factors and no general trend. For the first time in the Siebengebirge, triple infections were detected; one type of infection in 2007 and two different types of *Borrelia* combination in 2008. In 1987/89 no discrimination between single and multiple *Borrelia* infections were carried out. Initially, more single than double infections of ticks with *Borrelia* were reported for different sites in Europe [18,75,76]. However, not only in the Siebengebirge, but also in Ireland and Denmark the percentages of mixed infections increased, and even quadruple infections occurred [77,78]. Multiple infections can increase the risk of infections by Lyme disease since the chance of infections of a competent vector is increased.

### Comparison of *Borrelia* genospecies between 2001, 2007 and 2008

Estimations of the infection risk require not only a determination of the numbers of ticks and the infection rates with *B. burgdorferi* s.l., but also determinations of the genospecies. *B. afzelii, B. garinii* and *B. valaisiana* are the three most abundant species in Europe [29]. This is also the case in the Siebengebirge, but the prevalence of *B. burgdorferi* genospecies has changed during the few last years. In 2001, at all three sites the most prominent genospecies was *B. valaisiana* (infection rate of 43.1%) [15], whereas in 2007 *B. garinii* and *B. afzelii* were detected in every second tick, and in 2008 *B. garinii* was the dominant species. Also changes of low-abundant genospecies occurred within the two collection years of the present study: For example, in 2007 *B. burgdorferi* s.s. was only detected in 7 out of 137 infected ticks, but one year later this species was found in 35 out of 116 ticks. Also *B. valaisiana* was rarely found in 2007 but in 19.8% of *Borrelia* infections in 2008.

Differences in the genospecies composition were also evident between the three study sites. In the Fraxino-Aceretum pseudoplatani, *B. valaisiana* as dominant species in 2001 did not re-appear to this extent in the present study [15]. Only the Luzulo-Fagetum milietosum showed a stable dominance of *B. garinii* with similar infection rates in all years. In the Galio-Fagetum typicum, in 2001 *B. garinii* and *B. afzelii* predominated, in 2007 only *B. afzelii,* and in 2008 similar numbers of ticks were infected with *B. garinii*, *B. valaisana* and *B. afzelii*. Such differences in the distribution of genospecies seem to be caused by differences in the host cenosis [29] and be based on different competences of vertebrate hosts for the respective genospecies. *B. afzelii* is mainly found in rodents such as *Apodemus* sp.*,*and *B. garinii* and *B. valaisiana* are associated with birds [31]. The complement system of rodents completely lyses different genotypes of *B*. *garinii* and *B*. *valaisiana* but not *B*. *afzelii*[79]. Vice versa, in birds the complement system lyses *B*. *afzelii,* but not *B*. *garinii* and *B*. *valaisiana*[79]. Comparing the reservoir capacity of different birds, pheasants (*Phasianus colchicus*) and passerines such as the European blackbird (*Turdus merula*) and the American robin (*T. migratorius)* were positively associated with *Borrelia* infections [80-82]. Both, rodents and birds are competent reservoir hosts of *Borrelia burgdorferi* s.s. [32]. In the Siebengebirge in all study years, the two bird genospecies, *B*. *garinii* and *B*. *valaisiana,* together predominated. Thus, birds in the Siebengebirge seem to be the most successful reservoir host for *Borrelia,* a phenomenon that was also suggested for 2001 [15]. Passerine birds are widely distributed in the Siebengebirge, and thus they have a high impact on the density of ticks.

*B. lusitaniae* was detected for the first time in the Siebengebirge in 2007. We cannot exclude it for 2001 since DNA probes for *B. lusitaniae*, *B. spielmanii* and *B. bissettii* were not used in that year. However, only 4 out of 65 *Borrelia*-positive tick samples in 2001 reacted only with the complex specific *B. burgdorferi* s.l. probe [15]. Initially, this genospecies was classified as non-pathogenic for humans because this species was not detected in humans but only found in animal hosts [83,84]. However, serious symptoms of Lyme borreliosis were induced in mice infected by *B. lusitaniae*[85], and recently the first isolate of this genospecies was found in a woman suffering from chronic skin lesions in Portugal [28]. Thus, *B. lusitaniae* represents a new *Borrelia* genospecies with a new risk for visitors of the Siebengebirge to be infected with Lyme disease. *B. lusitaniae* was frequently present in ticks from Mediterranean countries such as Portugal (first record), Tunisia and Morocco [84,86,87]. It was also found in the Czech Republic, Poland, Slovakia, Moldavia, Ukraine, Spain, France, Switzerland and South Germany [36,75,83,88-91]. The recent identifications in Denmark and Sweden demonstrated the ability of this genospecies to establish even in northern Europe [77,92]. In the Siebengebirge*, B. lusitaniae* was found in 15 ticks (13 nymphs and one adult from the Luzulo-Fagetum milietosum and one from the Fraxino-Aceretum pseudoplatani). The latter site was near the Luzulo-Fagetum milietosum. Half of these ticks were co-infected with *B. garinii* and one tick with *B. valaisiana* indicating that birds may have introduced this species to the Siebengebirge; a similar observation was made in Switzerland [93]. Birds are considered as main reservoir hosts for *B. lusitaniae*[35], but sand lizards (*Lacerta agilis*) and common wall lizards (*Podarcis muralis*) were also infected with *B. lusitaniae* in Germany [36]. These two lizard species exist in the Siebengebirge, but they are rare and thus presumably less important for the distribution of *B. lusitaniae* in the Siebengebirge. Since almost all *B. lusitaniae* were detected in May and June of 2007 (apart from one infected tick in the Fraxino-Aceretum pseudoplatani in September) but not again in 2008, and since the distribution of *Borrelia* is linked to the migration of birds [31] future investigations in *Borrelia* transmission in the Siebengebirge should consider migratory birds as potential hosts of *Borrelia*. The maintenance of *B. lusitaniae* in the local bird fauna of the Siebengebirge is rather unlikely because in that case *B. lusitaniae* should have been detected frequently alongside with *B. garinii* and *B. valaisiana* in ticks. However, although recently Norte *et al*. [94] confirmed *B. lusitaniae* in questing *I. ricinus* they could never detect this *Borrelia* species in the local bird fauna in Portugal nor in migratory birds. Instead, two *Borrelia* isolates were identical to *B. lusitaniae* detected in mice skin in Portugal [95] and in ticks feeding on lizards from central Europe, Madeira and Portugal [36,96,97]. Thus, mice and lizards may maintain *B. lusitaniae*, and birds only play a minor, temporary role in the *B. lusitaniae* distribution. However, mice commonly occur in the Luzulo-Fagetum milietosum and Fraxino-Aceretum pseudoplatani and nevertheless *B. lusitaniae* was only detected for a short time in 2007. Furthermore, Amore *et al.*[98] found *B. lusitaniae* only in ticks feeding on lizards, but not in ticks feeding on mice and birds. Thus, the distribution and maintenance of *B. lusitaniae* remains unclear and further investigations are needed including the analysis of ticks feeding on mice, lizards and birds in the Siebengebirge.

## Conclusions

Over the last two centuries tick densities have increased in the Siebengebirge, a dense forested nature reserve providing excellent abiotic and biotic conditions for ticks, without changes of the biotopes by human activities. These increases were most likely favored by climatic conditions. Although *Borrelia* infection prevalences did not increase simultaneously with increasing tick densities in all biotopes, significantly higher multiple infections of ticks with *Borrelia* occurred in 2007 than in 2001; for the first time triple infections with *Borrelia* were detected in 2007 and 2008 in the Siebengebirge. Furthermore, a new *Borrelia* species, *B. lusitaniae,* has been introduced to the Siebengebirge. Thus, the risk for visitors, woodmen, hunters, farmers and animals of the nature reserve Siebengebirge of being exposed to tick bites increased strongly since 1987, however, the risk of being infected by Lyme disease did not increase consequently simultaneously. Nevertheless, the increase of multiple *Borrelia* infections in ticks may represent a new potential risk factor.

## Competing interests

The authors declare that they have no competing interests.

## Authors’ contributions

AS, GAS and LG drafted the manuscript. AS and GAS designed the study and AA collected the ticks in the Siebengebirge, Germany. AS and CB performed the tick examinations of the samples in 2007, and VH and ZV carried out all further experiments of samples from 2008 and all genotyping experiments. All authors read and approved the final manuscript.
